# Association of PET-based estradiol-challenge test for breast cancer progesterone receptors with response to endocrine therapy

**DOI:** 10.1038/s41467-020-20814-9

**Published:** 2021-02-02

**Authors:** Farrokh Dehdashti, Ningying Wu, Cynthia X. Ma, Michael J. Naughton, John A. Katzenellenbogen, Barry A. Siegel

**Affiliations:** 1 Alvin J. Siteman Cancer Center, Washington University School of Medicine, St. Louis, MO USA; 2 Division of Nuclear Medicine, Edward Mallinckrodt Institute of Radiology, Washington University School of Medicine, St. Louis, MO USA; 3 Division of Public Health Sciences, Department of Surgery Washington University School of Medicine, St. Louis, MO USA; 4 Division of Oncology, Department of Medicine, Washington University School of Medicine, St. Louis, MO USA; 5 Saint Francis Healthcare, Cape Medical Oncology, Cape Girardeau, MO USA; 6 Department of Chemistry and Cancer Center, University of Illinois, Urbana, IL USA

**Keywords:** Breast cancer, Breast cancer

## Abstract

Estrogen receptor (ER) testing of breast cancer imperfectly predicts response to endocrine therapy (ET). We hypothesize that a brief estradiol challenge will increase tumor progesterone receptor (PgR) levels only in tumors with functional ER. In this prospective, phase 2, single-center, single-arm trial (NCT02455453), we report the association of response to ET with change in tumor uptake of the progestin analog, 21-[^18^F]fluorofuranylnorprogesterone (FFNP), before and after a one-day estradiol challenge. In 43 postmenopausal women with advanced ER+ breast cancer, we show a post-challenge increase in tumor FFNP uptake only in 28 subjects with clinical benefit from ET (responders), but not in 15 without clinical benefit (nonresponders) (*p* < 0.0001), indicating 100% sensitivity and specificity. We further show significantly longer survival (*p* < 0.0001) in the responding subjects. Our results demonstrate that change in tumor FFNP uptake after estradiol challenge is highly predictive of response to ET in women with ER+ breast cancer.

## Abstract (web summary)

Clinical estrogen receptor (ER) testing for breast cancer is limited in predicting response to endocrine therapy (ET). In this phase 2 clinical trial, authors demonstrate that the responsiveness to ET can be predicted by use of PET/CT with 21-[^18^F]fluorofuranylnorprogesterone (FFNP) to detect the change in tumor progesterone receptor (PgR) levels after a one-day estradiol challenge.

## Introduction

Over 70% of patients with breast cancer have hormone-receptor-positive disease, characterized as being estrogen-receptor-positive (ER+), progesterone-receptor-positive (PgR+), or both^1,2^. ER positivity has significant prognostic value and is a main predictor of sensitivity to endocrine therapy (ET). However, up to 50% of ER+ breast cancers do not respond to ET^3^. There is an unmet clinical need to develop more precise predictive biomarkers.

PgR transcription is directly regulated by estrogen action through the ER, and the measurement of PgR levels was originally proposed as a way to identify ER+ tumors with functional ER capable of mediating ET response^4^. However, assessment of PgR by immunohistochemistry (IHC) has been limited by several factors, including variable standards for positivity and lack of reproducibility due to sampling errors^5^. More significantly, PgR levels are elevated only when ER is functional and estrogen is present; menopausal estrogen levels are likely insufficient to maximally elevate PgR levels^6^, although local levels of estradiol in the tumors themselves might be higher than circulating levels due to intratumoral estrogen biosynthesis^7^. These limitations contribute to inconsistent data observed in clinical studies assessing the value of PgR for predicting ET benefit. Clearly, better methods are needed to determine the quantity and functional status of tumor ER, as well as PgR, in order to reliably identify patients most likely to benefit from ET.

We developed a PgR-binding, progestin-analog radiopharmaceutical (21-[^18^F]fluorofuranylnorprogesterone [FFNP])^8^, and demonstrated significantly greater FFNP uptake in PgR+ than PgR– breast cancers by positron emission tomography (PET)^9^. Additionally, in mouse mammary tumors, we found that the increase in FFNP uptake after estrogen treatment was both rapid and robust^10^; similar observations were made by others in human breast cancer xenograft models^11,12^. We undertook this study to determine functional ER in patients with ER+ breast cancer by using FFNP-PET to measure the change in tumor PgR levels in vivo in response to a brief dosage of estradiol. In this work, we show that this PgR-based estradiol-challenge test, as an in vivo assessment of tumor ER functional status, would be a much stronger predictor of benefit from ET than the mere presence of ER and/or PgR by IHC in a biopsy sample of a single lesion.

## Results

### Patient demographics

Forty-seven women with ER+, human epidermal growth factor receptor 2-negative (HER2−) locally advanced, and locally recurrent or metastatic breast cancer were enrolled between June 2015 and December 2018 (see STARD flow diagram in Supplementary Fig. 1). Four subjects were excluded from the response analysis set: 1 declined ET, two had only hepatic metastatic disease (precluding assessment of tumor uptake because of intense FFNP uptake in normal liver), and 1 had tumor deposits with no definite uptake on either FDG ([^18^F]fluorodeoxyglucose)-PET/CT or FFNP-PET/CT. In the remaining 43 subjects, the median age was 60 years (range 46–82 years); 37 subjects (86%) had metastatic disease and six (14%) had either locally advanced primary cancers (*n* = 5) or locally recurrent disease (*n* = 1) (Table 1). Receptor status was determined for the original locally advanced primary tumor or a histologically proven recurrent or metastatic lesion in 21 subjects; in the remaining 22 subjects, receptor status of metastatic or recurrent disease was assumed to be the same as that of the original primary tumor. All tumors were ER+ and 27 were PgR+. Among the 43 subjects, 11 had soft tissue disease, 7 had soft tissue and bone disease, 6 had soft tissue and visceral disease, 2 had visceral-only disease, 9 had bone-only disease, 4 had bone and visceral disease, and 4 had soft tissue, bone and visceral disease. The imaging data were derived from 134 different sites of disease involving bone (*n* = 70), pleura (*n* = 2), omentum (*n* = 3), lymph nodes (*n* = 32), lung (*n* = 9), or breast and chest wall (*n* = 18).

**Table 1:** Patient characteristics by clinical benefit.

Characteristics	All	Nonresponders	Responders	*p*^a^
No. of patients, *n* (%)	43 (100%)	15 (65%)	28 (35%)	
Response at 6 months, *n* (%)				
Objective response	15 (35%)	0 (0%)	15 (54%)	
Stable disease	13 (30%)	0 (0%)	13 (46%)	
Progressive disease within 6 months	15 (35%)	15 (100%)	0 (0%)	
Age (years), median (Q1–Q3)^b^	60 (54–66)	61 (55–66)	58 (54–66)	0.6367
Race, *n* (%)				0.4508
Caucasian	33 (77%)	13 (87%)	20 (71%)	
African American	10 (23%)	2 (13%)	8 (29%)	
Disease status, *n* (%)				0.6427
Metastatic disease/chest wall recurrence	38 (88%)	14 (93%)	24 (86%)	
Locally advanced	5 (12%)	1 (7%)	4 (14%)	
PgR status, *n* (%)				0.7817
Negative	16 (37%)	6 (40%)	10 (36%)	
Positive	27 (63%)	9 (60%)	18 (64%)	
Heterogeneous tumor FFNP increase with estradiol challenge, *n* (%)				1.0000
No	33 (77%)	12 (80%)	21 (75%)	
Yes	10 (23%)	3 (20%)	7 (25%)	
Disease site, *n* (%)				0.1001
Bone	9 (21%)	4 (27%)	5 (18%)	
Soft tissue	11 (26%)	2 (13%)	9 (32%)	
Visceral	2 (5%)	2 (13%)	0 (0%)	
Bone & soft tissue	7 (16%)	1 (7%)	6 (21%)	
Bone & visceral	4 (9%)	2 (13%)	2 (7%)	
Soft tissue & visceral	6 (14%)	1 (7%)	5 (18%)	
Bone, soft tissue, & visceral	4 (9%)	3 (20%)	1 (4%)	
Disease site, *n* (%)				0.1094
Bone and/or soft tissue	27 (63%)	7 (47%)	20 (71%)	
Visceral and/or bone, soft tissue	16 (37%)	8 (53%)	8 (29%)	
Prior neoadjuvant therapy, *n* (%)				0.4581
None	34 (79%)	13 (87%)	21 (75%)	
1 Regimen	9 (21%)	2 (13%)	7 (25%)	
Prior adjuvant therapy, *n* (%)				0.2275
None	16 (37%)	3 (20%)	13 (46%)	
1 Regimen	12 (28%)	5 (33%)	7 (25%)	
2+ Regimens	15 (35%)	7 (47%)	8 (29%)	
Prior metastatic therapy, *n* (%)				0.0078
None	25 (58%)	4 (27%)	21 (75%)	
1–2 Regimens	8 (19%)	5 (33%)	3 (11%)	
3+ Regimens	10 (23%)	6 (40%)	4 (14%)	

^a^Fisher’s exact test (if cell count is less than 5) or Chi-square test for categorical variable; Kruskal-Wallis test for continuous variable.

^b^Q1 is 25^th^ percentile, and Q3 is 75^th^ percentile. The range of age is 46–82 years.

Twelve subjects (28%) were treatment-naïve, but the majority had received various forms of prior systemic therapy, with most being ET-based regimens (Tables 1 and 2); 9 patients had neoadjuvant therapy, 27 had adjuvant therapy, and 18 had therapy in the metastatic setting. Following completion of PET studies, the subjects underwent various types of ET (Table 2).

**Table 2:** Treatment summary.

Prior treatment	
None	12
Chemotherapy alone	1
ET alone	4
ET and chemotherapy	11
ET, chemotherapy, and CDK 4/6 inhibitor	3
ET and CDK 4/6 inhibitor	4
ET, chemotherapy, and mTOR inhibitor	3
ET, CDK 4/6 inhibitor, and mTOR inhibitor	1
ET, chemotherapy, CDK 4/6 inhibitor, and AKT inhibitor	2
ET, mTOR inhibitor, and chemotherapy	1
ET, chemotherapy, mTOR inhibitor, and trastuzumab*	1
ET during study and response within 6 months	
Aromatase inhibitor (2 PD, 1 SD, 6 PR)	9
Tamoxifen and AKT inhibitor (1 SD)	1
Aromatase inhibitor and CDK 4/6 inhibitor (5 PD, 8 SD, 7 PR)	20
Fulvestrant and CDK 4/6 inhibitor (5 PD, 1 SD)	6
Aromatase inhibitor and mTOR inhibitor (3 PD)	3
Aromatase inhibitor, gonadotropin-releasing hormone agonist, and mTOR inhibitor (1 SD)	1
Aromatase inhibitor, CDK 4/6 inhibitor, and mTOR inhibitor (1 PR)	1
Aromatase inhibitor, gonadotropin-releasing hormone agonist (1 PR)	1
Aromatase inhibitor, CDK 4/6 inhibitor, and gonadotropin-releasing hormone agonist (1 PR)	1

*ET* endocrine therapy*, PD* progressive disease, *SD* stable disease, *PR* partial response, *AKT* protein kinase B.

^*^Patient had ER+/PR–/HER2+ primary breast cancer and developed ER+/PR+/HER2–metastatic disease several years later.

### Adverse events

There were no definite adverse or clinically detectable pharmacological effects associated with FFNP administration, and no significant changes in vital signs were observed. However, with estradiol administration, 11 subjects reported transient grade 1 (diarrhea 3, nausea 3, vomiting 1, oral dysesthesia 1, fatigue 2, musculoskeletal pain 3, headache 2, paresthesia 1) and grade 2 (vomiting 1, cramping 1, back pain 1) adverse effects; these may have been related to estradiol and subsided before or shortly after the second FFNP administration.

### Response to endocrine therapies

**Fig. 1:**
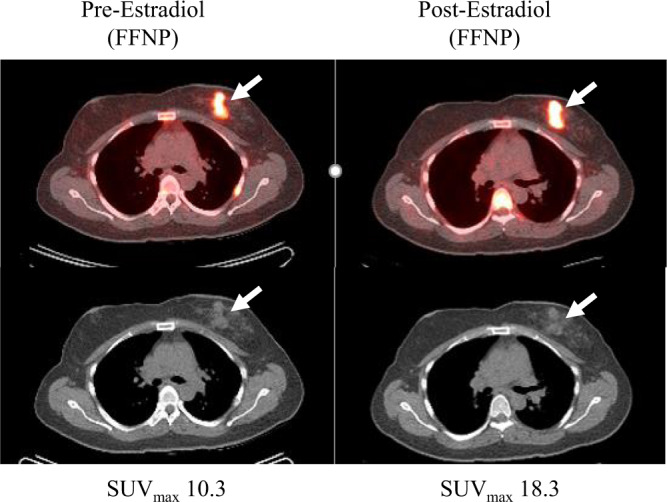
Responder. A woman with newly diagnosed ER+/PR+/HER2− invasive ductal carcinoma with metastatic disease at diagnosis in left axillary lymph nodes and a rib. She was treated with an aromatase inhibitor and palbociclib after the estradiol challenge test and had marked improvement of all lesions. Selected fused transaxial 21-[^18^F]fluorofuranyl-norprogesterone [FFNP]-PET/CT (top) and CT (bottom) images at baseline show intense FFNP uptake in the primary left breast cancer (arrows). One day after estradiol, tumor FFNP uptake measured as the maximum standardized uptake value (SUV_max_) (arrows) was increased by 78% from baseline.

**Fig. 2:**
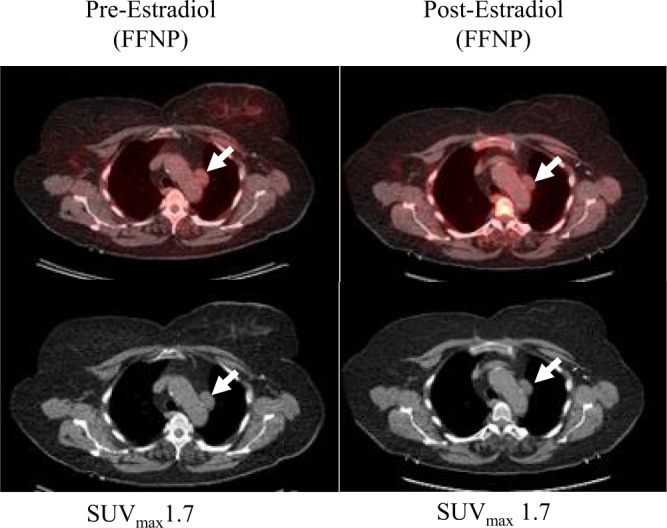
Nonresponder. A woman with ER+/PR−/HER2− invasive ductal carcinoma post neoadjuvant ET and breast conserving surgery, who developed metastatic disease to left prevascular and aortopulmonary lymph nodes. She was treated with an aromatase inhibitor after the estradiol challenge test but developed progressive disease. Selected fused transaxial FFNP-PET/CT (top) and CT (bottom) images at baseline show minimal FFNP uptake in the prevascular lymph node metastasis (arrows). One day after estradiol, tumor FFNP uptake measured as the maximum standardized uptake value (SUV_max_) (arrows) is unchanged.

Fifteen subjects (35%) were nonresponders and experienced disease progression within 6 months of initiating ET (Table 1). Twenty-eight subjects (65%) were responders and experienced clinical benefit (no disease progression within 6 months), including 15 (54%) with partial response and 13 (46%) with stable disease (Figs. 1 and 2).

**Fig. 3:**
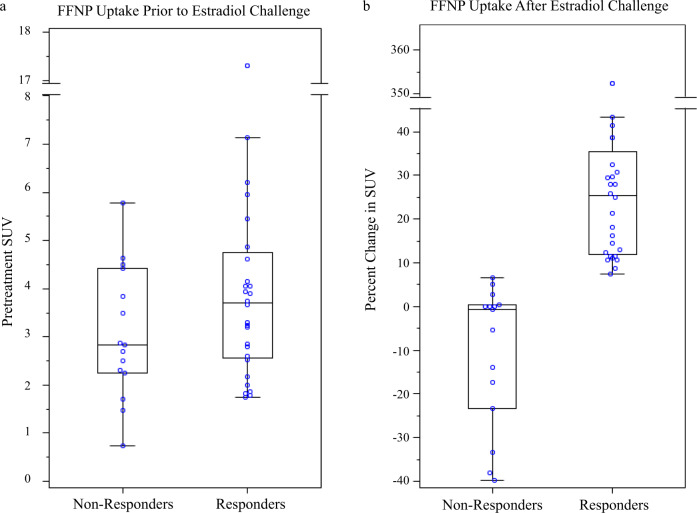
Tumor FFNP uptake in responders and nonresponders. **a** Baseline SUV, **b** Percent change in SUV after estradiol challenge.

**Fig. 4:**
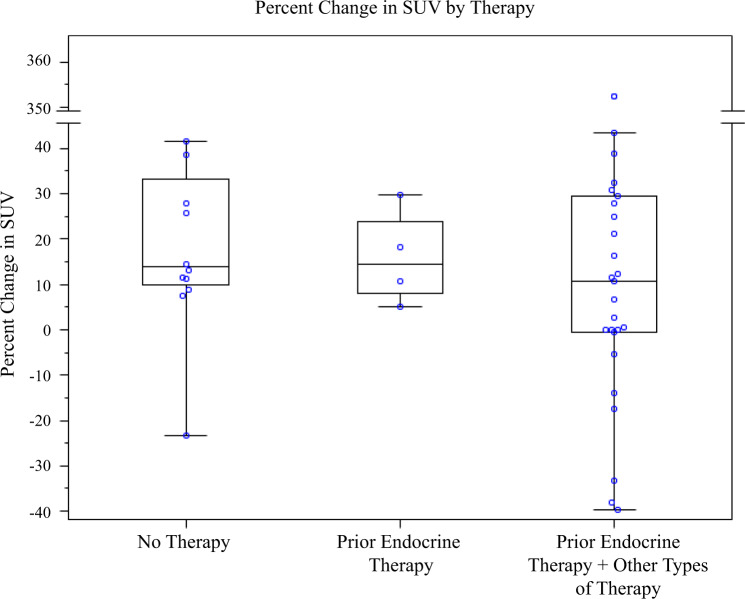
Percent change in tumor FFNP uptake with estradiol challenge based on prior therapies. Relationship of percent change in standardized uptake value (SUV) for FFNP after estradiol challenge to previous subject therapy.

Baseline tumor FFNP uptake did not differ significantly between responding and nonresponding subjects (median (Q1–Q3): 3.7 (2.6–4.7) versus 2.8 (2.3–4.4), *p* = 0.18) (Table 3). Following estradiol challenge, however, tumor FFNP uptake was significantly increased compared to baseline in responding subjects but not in nonresponding subjects, and it actually decreased relative to baseline in many of the latter (*p* < 0.0001) (Fig. 3 and Supplementary Table 1). The median percentage change in tumor FFNP uptake in responding subjects was 25.4% (Q1–Q3: 11.9–35.5%); and in nonresponding subjects it was −0.7% (Q1–Q3:−23.4% to 0.4%). Figure 3 shows the complete separation based on the percentage change of FFNP standardized uptake values (SUV) in responding and nonresponding subjects. That is, the minimum value of percentage change in SUV (i.e., 7.4) among responders was greater than the maximum value of percentage change in SUV (i.e., 6.7) among nonresponders. Using any value of the percentage change in SUV between 6.7 and 7.4 (e.g., 7) as a cutoff to indicate clinical benefit results in 100% sensitivity, specificity, positive-predictive value (PPV), and negative-predictive value (NPV). The percentage change of SUV did not differ significantly across different types of prior therapy (*p* = 0.47) (Fig. 4).

**Table 3:** Summary of FFNP-PET findings.

	Clinical effect of endocrine therapy		
	Response (*n* = 28) Median (Q1–Q3)^a^	No response (*n* = 15) Median (Q1–Q3)	*p*^b^
FFNP uptake prior to estradiol challenge			
Pretreatment SUV^c^	3.7 (2.6–4.7)	2.8 (2.3–4.4)	0.18
FFNP uptake after estradiol challenge			
Percentage change in SUV	25.4 (11.9–35.5)	−0.7 (−23.4 to 0.4)	<0.0001

^a^Q1 = 25^th^ percentile, Q3 = 75^th^ percentile.

^b^Kruskal-Wallis test.

^c^SUV = standardized uptake value.

**Fig. 5:**
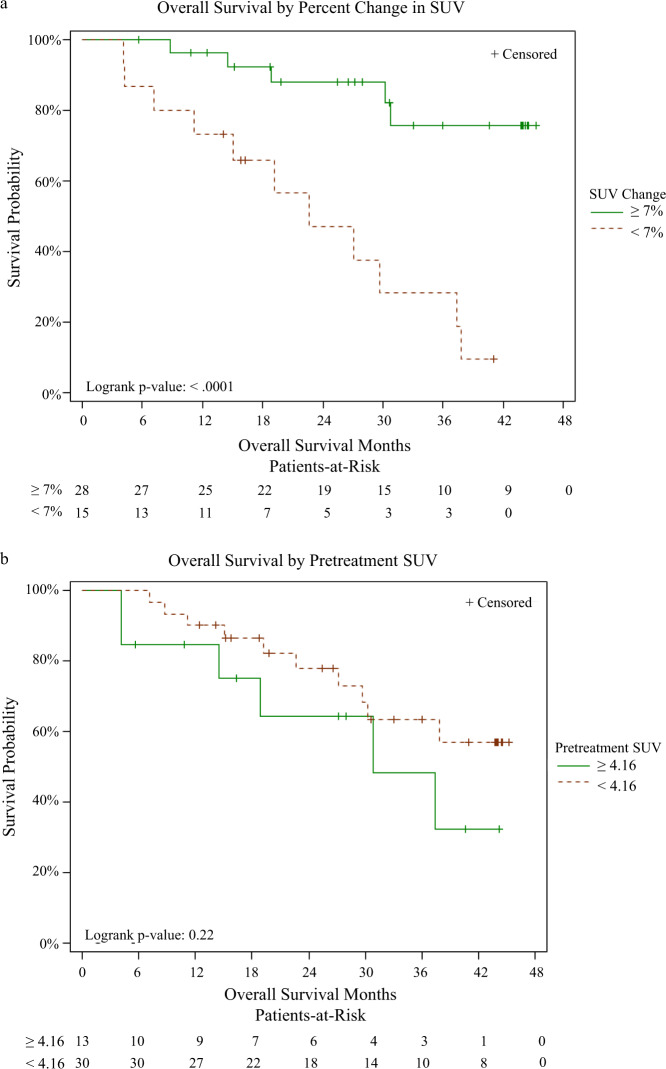
Overall survival results. **a** Dichotomized based on percent change in standardized uptake value (SUV) for FFNP after estradiol challenge (≥7% vs. <7%). The overall survival results based on clinical response (responders versus nonresponders) were identical to those based on percent change in FFNP SUV. **b** Dichotomized based on the optimal cut-off value of the baseline FFNP SUV (≥4.16 vs. <4.16).

As shown in Fig. 3, all of the 28 responders had a SUV change ≥7% and all of the 15 nonresponders had a SUV change <7%. Accordingly, the Kaplan-Meier overall survival (OS) curve based on clinical benefit and that based on the percentage change in the FFNP SUV following estradiol were identical (Fig. 5a). Clinical responders (subjects with SUV change ≥7%) had a significantly longer survival (*p* < 0.0001) than clinical nonresponders (subjects with SUV change <7%). With a median follow-up of 27.1 months, the estimated median survival was 22.6 months in nonresponding subjects (95% CI 7.1–37.4 months), and the median survival has not been reached among responding subjects. The baseline tumor FFNP uptake was not significantly associated with OS (*p* = 0.22) (Fig. 5b), when dichotomized at an optimal cut-point of 4.16, which was determined by the log-rank test statistic with an adjustment for bias^13^ to result in a most significant split.

As shown in Table 1, unlike the predictive value of the change in tumor FFNP uptake, other features were not associated with clinical benefit, except for prior metastatic disease therapy (*p* = 0.0078). Further subgroup analysis showed that the SUV change still can significantly distinguish nonresponders and responders among patients with prior metastatic disease therapy (*n* = 18, *p* = 0.0005) and among those without such therapy (*n* = 25, *p* = 0.0019).

Intra-subject tumor uptake change after estradiol challenge was homogeneous in 33 subjects (77%) and heterogeneous (defined as any increase in uptake in one or more lesions, while one or more other lesions had decreased or no change in uptake) in 10 (23%), occurring in 7 (25%) responding and 3 (20%) nonresponding subjects (*p* = 1.0 for association with clinical benefit) (Table 1). The tumors were PgR+ in 18 (64%) of 28 responding and 9 (60%) of 15 nonresponding subjects (*p* = 0.78). There was no significant association between clinical benefit and serum estradiol level at baseline and after estradiol challenge (*p* = 0.37 and *p* = 0.25, respectively) (Table 4a). In addition, there was no significant association between change in tumor FFNP uptake and serum estradiol level at baseline and after estradiol challenge (*p* = 0.14 and *p* = 0.17, respectively) (Table 4b).

**Table 4:** . Relationship of serum estrogen level and response (a) and change in FFNP uptake after estradiol challenge (b).

(a) Relationship between serum estrogen level and clinical response (*n* = 37)				
	Clinical effect of endocrine therapy			
	Response (*n* = 24) Median (Q1–Q3)^a^	No response (*n* = 13) Median (Q1–Q3)		*p*^b^
Prior to estradiol challenge				
Baseline estradiol level	15.1 (13.4–19.1)	13.3 (8.6–19.3)		0.37
After estradiol challenge				
Change of estradiol against baseline^c^	9.9 (5.8–15.5)	13.2 (10.2–21.8)		0.25

(b) Relationship between serum estradiol level and FFNP uptake change (*n* = 37)				
	FFNP uptake change			
	Spearman correlation coefficients			*p*^d^
Prior to estradiol-challenge test				
Baseline estradiol level	0.25			0.14
After estradiol-challenge test				
Change rate of estradiol level^c^	−0.23			0.17

^a^Q1 = 25^th^ percentile, Q3 = 75^th^ percentile.

^b^Kruskal-Wallis test.

^c^Calculated as (estradiol level after estradiol challenge − baseline estradiol level)/baseline estradiol level.

^d^Spearman-Brown test.

## Discussion

We found that an estradiol-challenge test that monitors an increase in tumor PgR by FFNP-PET can rapidly and accurately predict response or lack thereof to subsequent ET in patients with advanced ER+ breast cancer. Most of our subjects (72%) had received prior systemic therapy (Table 2). The type of previous therapy did not affect changes in FFNP uptake after estradiol challenge (Fig. 4), and a heterogeneous response to estradiol had no significant association with clinical benefit (*p* = 1.0). In addition, there was not a significant correlation between serum estradiol level, at baseline or after estradiol challenge, and the change in tumor FFNP after estradiol challenge or clinical benefit (Table 4). All subjects with a > 6.7% increase in FFNP uptake after estradiol challenge, classified as having functional ER, were subsequently identified as responders (100% PPV and NPV). They also had a significantly longer OS than did those with a decrease in FFNP or an increase ≤6.7%, identical to that of subjects who were subsequently identified as nonresponders (Fig. 5a).

Of note, most of our subjects (28 of 43) received CDK4/6 inhibitor/ET combination therapy, and the test identified all 10 nonresponders (Table 2). This suggests that functional ER could also be important for the efficacy of CDK4/6 inhibitors in ER+ breast cancer. This is consistent with the preclinical observation that CDK4/6 inhibitors are preferentially effective in luminal breast cancer^14^. Further evaluation of the estradiol-challenge test with FFNP-PET in predicting CDK4/6 inhibitor efficacy in ER+ breast cancer is warranted.

Our original hormone-challenge test was developed when tamoxifen was the preferred ET agent, and it became known that some patients who ultimately responded to tamoxifen experienced a transient increase in pain symptoms 7–10 days after initiating tamoxifen therapy. This tamoxifen clinical flare was ascribed to an initial partial agonist activity of tamoxifen^15^, but was not a reliable predictor of ET response^16^. By monitoring tumor metabolism with FDG-PET, at baseline and after 7–10 days of tamoxifen, we could detect a tamoxifen metabolic flare (increase in tumor SUV on the post-tamoxifen FDG-PET) that was highly predictive of benefit from continued tamoxifen therapy in largely treatment-naïve patients (PPV = 91%; NPV = 94%). These values were marginally greater than those based on 16α-[^18^F]fluoroestradiol (FES)-PET for detecting ER at baseline^17^. When aromatase inhibitors replaced tamoxifen as ET, we replaced the 7–10 days tamoxifen challenge with the current one-day estradiol challenge, which was followed by treatment with aromatase inhibitors or fulvestrant^18^. In more heavily pretreated subjects, we also found high predictive values for ET benefit from a post-estradiol increase in tumor FDG uptake (PPV = 100%; NPV = 94%), with greater predictive accuracy in this subject cohort than baseline FES-PET (PPV = 50%; NPV = 81%).

Despite good predictive values, the percentage change in FDG uptake and separation between ET responders and nonresponders in these FDG-PET-based hormone-challenge studies was relatively small. In addition, for FDG-PET/CT, patients need to fast to control blood glucose and insulin levels. By contrast, the estradiol-challenge test using FFNP-PET to measure changes in PgR levels gave markedly greater changes in tracer uptake, with complete separation between ET responders and nonresponders in a more diverse cohort of patients. Also, by using PgR as a biomarker, there is no need to monitor and control blood glucose levels.

Prior to this clinical study, we evaluated changes in PgR levels monitored by FFNP-PET in an ER+, hormone-responsive mouse mammary cancer model^10,19^. We found large increases in FFNP uptake after administration of estradiol and large decreases in FFNP uptake after oophorectomy or administration of fulvestrant in ovary-intact mice. Thus, in this mouse model, changes in FFNP uptake accurately predicted sensitivity to estrogen addition or ablation therapy. Accordingly, in principle, it also should be possible clinically to test the hormone sensitivity of breast cancer with FFNP-PET in order to monitor a decrease in PgR levels after a period of estrogen deprivation or ER blockade. Most breast cancer patients, however, are elderly, so their basal PgR levels should be low, reflecting low menopausal estrogen levels. Thus, the dynamic range for a downward change in PgR level from estrogen deprivation or blockade in elderly patients is likely to be less than that for the upward change we have monitored with the estradiol-challenge test. A test for downward change in PgR levels to predict ET responsiveness would be more feasible in premenopausal breast cancer patients. It is of note that, 2 weeks after initiating AI therapy, a decrease in the SUV for FDG was detected in some breast cancers and correlated with low levels of the proliferation marker Ki-67 at the time of the second image; the relation to therapy response, however, was not reported^20^.

While currently, selection of ET for women with advanced ER+ breast cancer is based on IHC assays of ER, PgR, and HER2, there are many emerging methods based on more extensive genomic, transcriptomic, proteomic, metabolomic, and other information-rich characterizations of biopsy samples^21–23^. These “-omic” analyses, as well as those based on specific candidate genes of interest on tumor tissue biopsies largely remain investigational. Assessment of Ki-67 by IHC following initiation of neoadjuvant ET may be a promising biomarker of endocrine sensitivity, but requires at least 2 weeks of treatment^24^. In addition, standardization of the Ki-67 analysis is required before its eventual clinical application for this purpose^25^. Some of these approaches may prove to have good predictive value for benefit from ET alone. It will take some time, however, to determine the accuracy of such tests, and they will be affected by the aforementioned concerns regarding sampling error and within-subject tumoral heterogeneity associated with needle biopsy, as well as intra-subject heterogeneity and biopsy-inaccessible lesions.

Our study has several limitations. It is a single-institution study with a relatively small sample size and with all imaging performed on a single PET/CT scanner. Despite the excellent separation of responders from nonresponders, assessment of the test–retest repeatability of measurements of FFNP uptake should be performed in future studies. Additionally, we designed this study to have post-estradiol PET/CT performed about 24 h after the last dosage of estradiol, but further study to find the optimal timing will be necessary.

FFNP-PET before and after administration of estradiol over 24 h can be accomplished in as little as 2 days before ET is started and discriminates likely responders from nonresponders with high accuracy, thus allowing for risk stratification equivalent to that obtained by much longer clinical observations alone. While the results of this study are extremely promising, our findings need to be confirmed in a multicenter trial before this method can be used to guide ET in clinical practice.

## Methods

### Study design and participants

This was a prospective, single-center, single-arm trial (NCT02455453). Postmenopausal women with ER+, HER2–, and locally advanced, locally recurrent or metastatic breast cancer were eligible for participation if they had measurable or evaluable disease by Response Evaluation Criteria in Solid Tumors (RECIST 1.1), had ECOG performance status 0–2, and were to be treated with ET. ER positivity was confirmed by IHC on the primary breast cancer or on a recurrent or metastatic lesion in all subjects. HER2 was considered negative if scored 0 or +1 by IHC or if the HER2/CEP17 ratio was <2.0 by FISH. The study was performed under physician-sponsored IND 76,214 and was approved by the Institutional Review Board and the Radioactive Drug Research Committee of Washington University School of Medicine, as well as by the Protocol Review and Monitoring Committee of the Alvin J. Siteman Cancer Center. All subjects gave written informed consent for study participation. The study was conducted in accordance with the Declaration of Helsinki and the International Council on Harmonization Guidelines for Good Clinical Practice.

All subjects underwent standard clinical evaluation, including complete history and physical examination, complete blood count, liver function studies, and necessary radiological examinations, e.g., CT of the chest, abdomen and pelvis, skeletal scintigraphy, and FDG-PET/CT, as medically indicated. After initiation of ET, the treating medical oncologist, who was blinded to the results of FFNP-PET, evaluated subjects every 3 months (or earlier, if necessitated by symptoms or other evidence of progression) until disease progression. The protocol did not mandate specific timing of follow-up assessments, but it is routine clinical practice at our institution to have imaging every 3–6 months during therapy. Clinical benefit (defined below) was determined 6 months after beginning ET.

### PET imaging and estradiol-challenge test

FFNP was prepared using an adaptation of a published procedure^8^. All subjects underwent two FFNP-PET/CT studies on 2 separate days, with imaging generally extending from the skull base to the upper thighs (other body parts were included, as applicable based on known sites of disease). FFNP (median dosage 9.2 mCi, range 3.9–10.6 mCi) was injected intravenously and imaging with a CTI/Siemens Biograph 40 HD PET/CT scanner began approximately 40 min later. Following a CT for attenuation correction, emission imaging was obtained (2–5 min per bed position, adjusted for subject height, weight, and injected FFNP dosage). Scans were reconstructed at a 5-mm slice thickness. FFNP dosage and scan parameters were matched as closely as possible for both FFNP-PET/CT scans in each subject.

For safely evaluation, all subjects had vital signs measured within 30 min before injection of FFNP, within 30 min after injection, and at the completion of each imaging session. A follow-up telephone call was made to the subject 24 ± 6 h post injection to assess for adverse events.

The estradiol challenge consisted of a total dosage of 6 mg, administered orally as one 2-mg tablet approximately every 8 h for 3 doses within a 24-h period. The final estradiol dose was taken at a median of 27.9 h (range 6.9–46 h) before injection of FFNP for the second PET/CT. The 6-mg dosage is the currently recommended daily dosage for estradiol treatment of metastatic breast cancer^26,27^. Serum estradiol level was assessed with a high-sensitivity radioimmunoassay before each of the two FFNP injections^28^ to document the post-challenge change in estradiol level.

### Response criteria

Response was determined in accordance with RECIST 1.1 for patients with measurable disease^29^. For subjects with bone-dominant or bone-only disease, response was evaluated by both imaging, including serial bone scintigraphy in conjunction with assessment of FDG-PET/CT findings or other imaging studies if performed, and clinical criteria, including tumor markers and symptoms. In these patients, a complete response was defined as disappearance of all objective and clinical disease, including complete normalization of radiological studies and tumor markers. A partial response was defined as a decrease in pain with evidence of re-calcification of known osseous lesions on radiography. Disease progression was defined as worsening of disease on anatomical and/or functional imaging or worsening of pain and decline in performance status. Any response that did not meet the criteria for complete response, partial response, or progression was defined as stable disease. For analysis of the prediction of response based on FFNP-PET findings, response was dichotomized as follows: subjects who derived clinical benefit, defined as achieving objective response or stable disease for at least 6 months, were considered responders and those with progressive disease within 6 months as nonresponders.

### Data analysis

All FFNP-PET/CT images were evaluated semi-quantitatively by determination of the maximum standardized uptake values (SUV_max_) of tumor foci. The percentage changes in SUVs for FFNP were recorded. In subjects with multiple lesions, the SUVs of up to five lesions (typically, the most intense lesions) were determined and the overall average values for all selected lesions in a given subject were recorded and used in comparisons with clinical response. To evaluate for intra-subject heterogeneous response to estradiol (defined as any increase in uptake in one or more lesions, while one or more other lesions had decreased or no change in uptake), the change in FFNP uptake after estradiol in individual lesions was compared to baseline uptake.

### Power analysis

We assumed an ET response rate of 20–50%. Using a two-sided independent *t*-test with 80% power at a 0.05 significance level, a sample of 10 responders versus 40 nonresponders (i.e., 20% response rate) could allow us to detect a minimum of 101% SD between-group difference in terms of percentage changes in FFNP uptake after estradiol challenge, where SD represents a pooled standard deviation of the FFNP uptake changes among both responders and nonresponders. A sample of 25 responders versus 25 nonresponders (i.e., 50% response rate) could allow us to detect a minimum of 80.9% SD between-group difference.

### Statistical analysis

SAS version 9.4 (SAS Institute, Cary, NC) was used for all statistical analyses. Demographic and clinical characteristics were summarized by descriptive statistics. Associations were examined either by Kruskal-Wallis test/Spearman-Brown test for continuous variables or Chi-square test/Fisher exact test for categorical variables. Overall survival (OS) was defined as the duration of time (in months) from the date of baseline PET to death from any cause. Subjects with no death date were censored at the last follow-up date. Kaplan-Meier curves were used to display OS difference by clinical response or dichotomized by FFNP uptake (baseline or percentage change after estradiol challenge) and examined by the log-rank test. The optimal cut-point of baseline FFNP uptake that gave the maximum OS difference between the subjects with high and low SUV was determined by the log-rank test statistic with an adjustment for bias^13^.

### Reporting summary

Further information on research design is available in the Nature Research Reporting Summary linked to this article.

## Supplementary information

Supplementary information

Reporting summary

## Data Availability

The data that support the findings of this study are available within this article and its supplementary files or from the authors upon reasonable request. All data and code used in the production of this manuscript are available upon request to the corresponding author.
