# Supplementary material for: Association of PET-based estradiol-challenge test for breast cancer progesterone receptors with response to endocrine therapy
Source: Nat Commun. 2021 Feb 2;12:733. doi: 10.1038/s41467-020-20814-9 (PMC7854611; doi:10.1038/s41467-020-20814-9)
Supplement: Supplementary file 1 — Supplementary information [file 41467_2020_20814_MOESM1_ESM.pdf]

## Supplemental Figure 1. STARD Flow Diagram

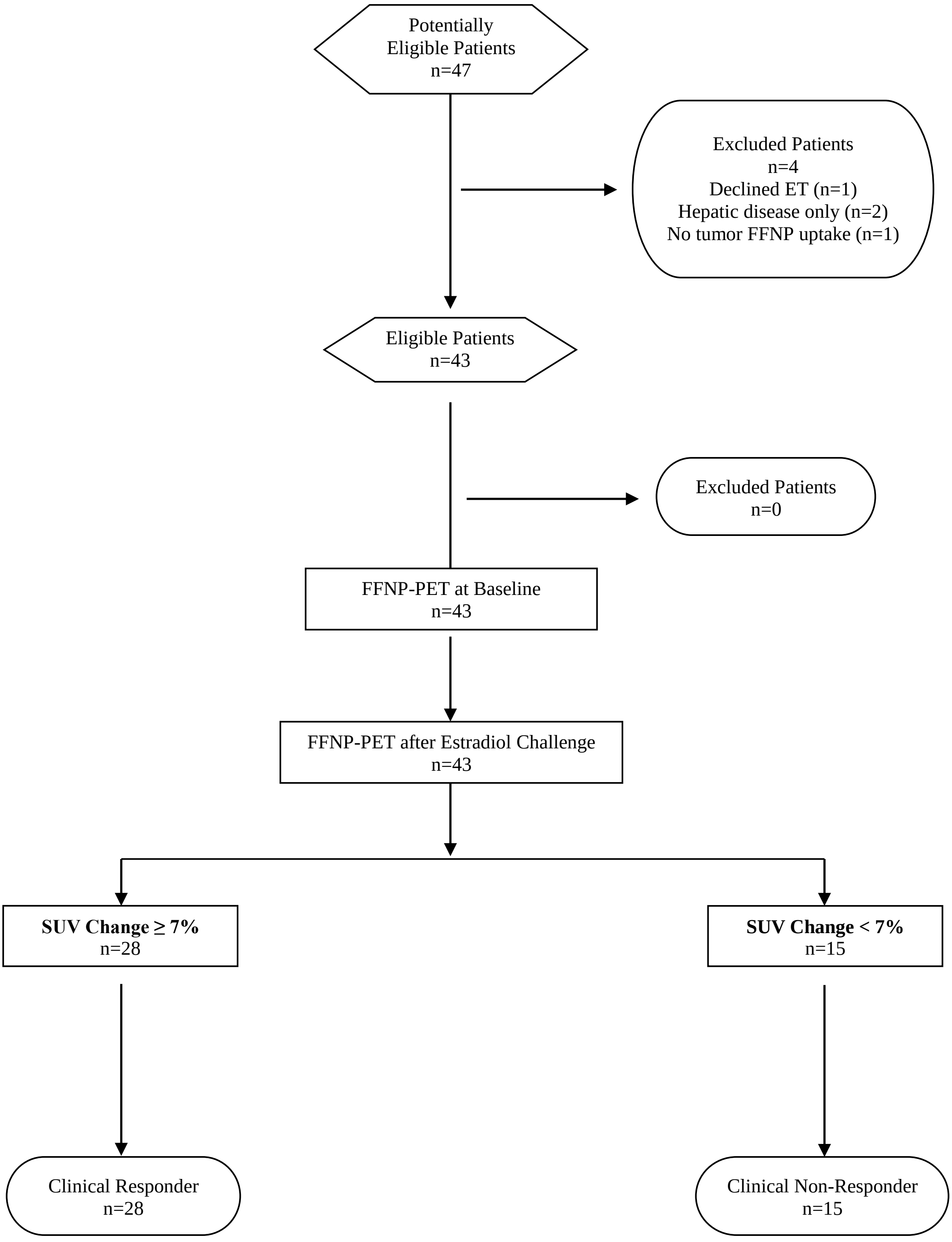

## Supplemental Table 1: Mean of $SUV_{max}$ Before and After Estradiol Challenge (Ordered by Increasing Percent Change)

| | | FFNP SUV Overall Average of Selected Lesions | | | |
|---|---|---|---|---|---|
| **Obs** | **Clinical Response** | **Pre Estradiol Challenge** | **Post Estradiol Challenge** | **Pre and Post Difference** | **% Change** |
| **1** | Non-Responders | 5.79 | 3.48 | -2.31 | -39.79 |
| **2** | Non-Responders | 2.83 | 1.75 | -1.08 | -38.16 |
| **3** | Non-Responders | 4.43 | 2.95 | -1.48 | -33.33 |
| **4** | Non-Responders | 3.85 | 2.95 | -0.90 | -23.38 |
| **5** | Non-Responders | 2.30 | 1.90 | -0.40 | -17.39 |
| **6** | Non-Responders | 2.87 | 2.47 | -0.40 | -13.95 |
| **7** | Non-Responders | 2.26 | 2.14 | -0.12 | -5.31 |
| **8** | Non-Responders | 0.73 | 0.73 | 0.00 | -0.68 |
| **9** | Non-Responders | 2.70 | 2.70 | 0.00 | 0.00 |
| **10** | Non-Responders | 2.50 | 2.50 | 0.00 | 0.00 |
| **11** | Non-Responders | 1.70 | 1.70 | 0.00 | 0.00 |
| **12** | Non-Responders | 4.64 | 4.66 | 0.02 | 0.43 |
| **13** | Non-Responders | 4.50 | 4.62 | 0.12 | 2.67 |
| **14** | Non-Responders | 1.47 | 1.55 | 0.08 | 5.10 |
| **15** | Non-Responders | 3.50 | 3.73 | 0.23 | 6.67 |
| **16** | Responders | 4.62 | 4.96 | 0.34 | 7.36 |
| **17** | Responders | 7.15 | 7.78 | 0.63 | 8.82 |
| **18** | Responders | 3.75 | 4.15 | 0.40 | 10.67 |
| **19** | Responders | 2.80 | 3.10 | 0.30 | 10.71 |
| **20** | Responders | 4.05 | 4.50 | 0.45 | 11.11 |
| **21** | Responders | 5.96 | 6.64 | 0.68 | 11.41 |
| **22** | Responders | 2.60 | 2.90 | 0.30 | 11.54 |
| **23** | Responders | 4.87 | 5.47 | 0.60 | 12.33 |
| **24** | Responders | 17.30 | 19.55 | 2.25 | 13.01 |
| **25** | Responders | 1.83 | 2.10 | 0.27 | 14.55 |
| **26** | Responders | 1.79 | 2.08 | 0.29 | 16.20 |
| **27** | Responders | 3.30 | 3.90 | 0.60 | 18.18 |
| **28** | Responders | 4.05 | 4.91 | 0.86 | 21.26 |
| **29** | Responders | 3.20 | 4.00 | 0.80 | 25.00 |
| **30** | Responders | 1.75 | 2.20 | 0.45 | 25.71 |
| **31** | Responders | 4.16 | 5.32 | 1.16 | 27.88 |
| **32** | Responders | 3.93 | 5.03 | 1.10 | 27.97 |
| **33** | Responders | 5.45 | 7.05 | 1.60 | 29.36 |
| **34** | Responders | 2.52 | 3.27 | 0.75 | 29.63 |
| **35** | Responders | 3.90 | 5.10 | 1.20 | 30.77 |
| **36** | Responders | 1.99 | 2.64 | 0.65 | 32.41 |
| **37** | Responders | 2.85 | 3.95 | 1.10 | 38.60 |
| **38** | Responders | 6.20 | 8.60 | 2.40 | 38.71 |
| **39** | Responders | 2.18 | 3.08 | 0.90 | 41.38 |
| **40** | Responders | 3.23 | 4.63 | 1.40 | 43.30 |
| **41** | Responders | 9.50 | 18.15 | 8.65 | 91.05 |
| **42** | Responders | 1.87 | 5.47 | 3.60 | 192.86 |
| **43** | Responders | 3.68 | 16.63 | 12.95 | 352.38 |
