# Supplementary material for: Association of PET-based estradiol-challenge test for breast cancer progesterone receptors with response to endocrine therapy
Source: Nat Commun. 2021 Feb 2;12:733. doi: 10.1038/s41467-020-20814-9 (PMC7854611; doi:10.1038/s41467-020-20814-9)
Supplement: Supplementary file 2 — Reporting summary [file 41467_2020_20814_MOESM2_ESM.pdf]

nature research

Corresponding author(s): DBPR NCOMMS-20-17888-T

Last updated by author(s): May 19, 2020

Reporting Summary

Nature Research wishes to improve the reproducibility of the work that we publish. This form provides structure for consistency and transparency in reporting. For further information on Nature Research policies, see our Editorial Policies and the Editorial Policy Checklist.

Statistics

For all statistical analyses, confirm that the following items are present in the figure legend, table legend, main text, or Methods section.

n/a	Confirmed	
☐	☒	The exact sample size (n) for each experimental group/condition, given as a discrete number and unit of measurement
☐	☒	A statement on whether measurements were taken from distinct samples or whether the same sample was measured repeatedly
☐	☒	The statistical test(s) used AND whether they are one- or two-sided<br>*Only common tests should be described solely by name; describe more complex techniques in the Methods section.*
☒	☐	A description of all covariates tested
☐	☒	A description of any assumptions or corrections, such as tests of normality and adjustment for multiple comparisons
☐	☒	A full description of the statistical parameters including central tendency (e.g. means) or other basic estimates (e.g. regression coefficient) AND variation (e.g. standard deviation) or associated estimates of uncertainty (e.g. confidence intervals)
☒	☐	For null hypothesis testing, the test statistic (e.g. F, t, r) with confidence intervals, effect sizes, degrees of freedom and P value noted<br>*Give P values as exact values whenever suitable.*
☒	☐	For Bayesian analysis, information on the choice of priors and Markov chain Monte Carlo settings
☒	☐	For hierarchical and complex designs, identification of the appropriate level for tests and full reporting of outcomes
☒	☐	Estimates of effect sizes (e.g. Cohen's d, Pearson's r), indicating how they were calculated

Our web collection on statistics for biologists contains articles on many of the points above.

Software and code

Policy information about availability of computer code

Data collection	n/a
Data analysis	SAS version 9.4 (SAS Institute, Cary, NC) was used for all statistical analyses.

For manuscripts utilizing custom algorithms or software that are central to the research but not yet described in published literature, software must be made available to editors and reviewers. We strongly encourage code deposition in a community repository (e.g. GitHub). See the Nature Research guidelines for submitting code & software for further information.

Data

Policy information about availability of data

All manuscripts must include a data availability statement. This statement should provide the following information, where applicable:

- Accession codes, unique identifiers, or web links for publicly available datasets
- A list of figures that have associated raw data
- A description of any restrictions on data availability

Raw data for SUVs and changes in SUVs are shown in Figures 3 and 4 and those for survival are shown in Figure 5.

Field-specific reporting

Life sciences study design

All studies must disclose on these points even when the disclosure is negative.

Sample size	We assumed an ET response rate of 20-50%. Using a 2-sided independent t-test with 80% power at a 0.05 significance level, a sample of 10 responders versus 40 nonresponders (i.e., 20% response rate) could allow us to detect a minimum of 101% SD between-group difference in terms of percent changes in FFNP uptake after estradiol challenge, where SD represents a pooled standard deviation of the FFNP uptake changes among both responders and nonresponders. A sample of 25 responders versus 25 nonresponders (i.e., 50% response rate) could allow us to detect a minimum of 80.9% SD between-group difference.
Data exclusions	Four subjects were excluded from the response analysis set: 1 declined ET, two had only hepatic metastatic disease (precluding assessment of tumor uptake because of intense FFNP uptake in normal liver), and 1 had tumor deposits with no definite uptake on either FDG-PET/CT or FFNP-PET/CT.
Replication	n/a
Randomization	n/a
Blinding	The treating medical oncologist(s) who assessed response/clinical benefit were blined to the results of FFNP-PET/CT.

Reporting for specific materials, systems and methods

We require information from authors about some types of materials, experimental systems and methods used in many studies. Here, indicate whether each material, system or method listed is relevant to your study. If you are not sure if a list item applies to your research, read the appropriate section before selecting a response.

Materials & experimental systems

n/a	Involved in the study	
☒	☐	Antibodies
☒	☐	Eukaryotic cell lines
☒	☐	Palaeontology and archaeology
☒	☐	Animals and other organisms
☐	☒	Human research participants
☐	☒	Clinical data
☒	☐	Dual use research of concern

Methods

n/a	Involved in the study	
☒	☐	ChIP-seq
☒	☐	Flow cytometry
☒	☐	MRI-based neuroimaging

Human research participants

Policy information about studies involving human research participants

Population characteristics	Postmenopausal women with ER+, human epidermal growth factor receptor 2-negative (HER2–), locally advanced, metastatic or recurrent breast cancer were eligible for participation if they had measurable or evaluable disease by Response Evaluation Criteria in Solid Tumors (RECIST 1.1), had ECOG performance status 0-2, and were to be treated with ET. ER positivity was confirmed by IHC on the primary breast cancer or on a recurrent or metastatic lesion in all subjects. HER2 was considered negative if scored 0 or +1 by IHC or if the gene amplification was <2.0 by FISH.<br>All subjects were women and ranged in age from 46-82 years (median 60 years). Of the evaluable subjects, 33 were Caucasian and 10 were African American. Other demographic details are shown in Table 1 and Table 2.
Recruitment	Subjects who met the eligibility criteria were recruited by their treating medical oncologist (most often Drs. Naughton or Ma, who were co-investigators). The only potential selection bias was subject willingness to participate.
Ethics oversight	This study was approved by the Institutional Review Board and the Radioactive Drug Research Committee of Washington University School of Medicine, as well as by the Protocol Review and Monitoring Committee of the Alvin J. Siteman Cancer Center. All subjects gave written informed consent for study participation.

Note that full information on the approval of the study protocol must also be provided in the manuscript.

Clinical data

Policy information about clinical studies

All manuscripts should comply with the ICMJEguidelines for publication of clinical research and a completedCONSORT checklist must be included with all submissions.

Clinical trial registration	NCT02455453
Study protocol	The full trial protocol was included at the request of the associate editor following the manuscript submission.

Data collection	Subjects were enrolled between June 2015 and December 2018, were assessed per standard of care for clinical response after undergoing FFNP-PET/CT before and after estradiol challenge, and their records assessed thereafter for survival until the study was closed with the IRB.
Outcomes	The primary objective was to evaluate whether the change in tumor uptake of FFNP following a 1-day estradiol challenge differs among patients who respond to endocrine therapy (ET) versus those who do not respond. Secondary objectives were as follows:<br>-Determine the optimum cutoff value for change in tumor 18F-FFNP uptake after estradiol to distinguish responders from nonresponders.<br>-Evaluate whether the change in tumor uptake of FFNP can identify patients with hormone-sensitive disease who will respond to ET with greater sensitivity and selectivity than tumor PgR measured by IHC.<br>-Evaluate the heterogeneity of tumor FFNP uptake at baseline and after estradiol challenge in patients with multiple metastatic foci.<br>-Explore whether response is related to FFNP uptake heterogeneity.<br><br>The primary outcome measures therefore were change in tumor FFNP uptake (measured as the maximum SUV) by PET/CT before and after estradiol challenge and clinical benefit (defined as response or stable disease) at 6 months (vs. progression).<br><br>To evaluate for intra-subject heterogeneous response to estradiol (defined as any increase in uptake in one or more lesions, while one or more other lesions had decreased or no change in uptake), the change in FFNP uptake after estradiol in individual lesions was compared to baseline uptake., and again related to the outcomes of clinical benefit vs. progression.
